# Validation of sick leave measures: self-reported sick leave and sickness benefit data from a Danish national register compared to multiple workplace-registered sick leave spells in a Danish municipality

**DOI:** 10.1186/1471-2458-12-661

**Published:** 2012-08-15

**Authors:** Christina Malmose Stapelfeldt, Chris Jensen, Niels Trolle Andersen, Nils Fleten, Claus Vinther Nielsen

**Affiliations:** 1 Section of Social Medicine and Rehabilitation, Deparment of Public Health, Aarhus University, Aarhus, Denmark; 2 Public Health and Quality Improvement, Aarhus, Central Denmark Region, Denmark; 3 Section of Biostatistics, Department of Public Health, Aarhus University, Aarhus, Denmark; 4 Department of Community Medicine, Faculty of Health Sciences, University of Tromsø, Tromsø, Norway

**Keywords:** Agreement, Eldercare sector, Public transfer payment, Register data, Self-report, Sensitivity, Sick leave, Specificity, Validation, Workplace record

## Abstract

**Background:**

Previous validation studies of sick leave measures have focused on self-reports. Register-based sick leave data are considered to be valid; however methodological problems may be associated with such data. A Danish national register on sickness benefit (DREAM) has been widely used in sick leave research. On the basis of sick leave records from 3,554 and 2,311 eldercare workers in 14 different workplaces, the aim of this study was to: 1) validate registered sickness benefit data from DREAM against workplace-registered sick leave spells of at least 15 days; 2) validate self-reported sick leave days during one year against workplace-registered sick leave.

**Methods:**

Agreement between workplace-registered sick leave and DREAM-registered sickness benefit was reported as sensitivities, specificities and positive predictive values. A receiver-operating characteristic curve and a Bland-Altman plot were used to study the concordance with sick leave duration of the first spell. By means of an analysis of agreement between self-reported and workplace-registered sick leave sensitivity and specificity was calculated. Ninety-five percent confidence intervals (95% CI) were used.

**Results:**

The probability that registered DREAM data on sickness benefit agrees with workplace-registered sick leave of at least 15 days was 96.7% (95% CI: 95.6-97.6). Specificity was close to 100% (95% CI: 98.3-100). The registered DREAM data on sickness benefit overestimated the duration of sick leave spells by an average of 1.4 (SD: 3.9) weeks. Separate analysis on pregnancy-related sick leave revealed a maximum sensitivity of 20% (95% CI: 4.3-48.1).

The sensitivity of self-reporting at least one or at least 56 sick leave day/s was 94.5 (95% CI: 93.4 – 95.5) % and 58.5 (95% CI: 51.1 – 65.6) % respectively. The corresponding specificities were 85.3 (95% CI: 81.4 – 88.6) % and 98.9 (95% CI: 98.3 – 99.3) %.

**Conclusions:**

The DREAM register offered valid measures of sick leave spells of at least 15 days among eldercare employees. Pregnancy-related sick leave should be excluded in studies planning to use DREAM data on sickness benefit. Self-reported sick leave became more imprecise when number of absence days increased, but the sensitivity and specificity were acceptable for lengths not exceeding one week.

## Background

The past couple of decades have seen growing concern over sick leave in working populations in Western societies as a public health problem. Sick leave has a multi-factorial aetiology [1-3]. Several scientific approaches have therefore been used which may explain the variety of approaches used to measure sick leave within an epidemiological framework: frequency of sick leave spells per individual, the total length of absence during a specified period, incidence rate, cumulative incidence and duration of absence spells [4]. However, in light of the large number of studies on sick leave, it is remarkably that only few validation studies have been performed.

Among the four traditional sources (employer’s personnel files, insurance-based data, national social security registers and self-reported data) from which sick leave data are traditionally retrieved, register-based sick leave data is an option available only in few countries. Even where registers are available, self-reported sick leave data are usually more easily acquired than data from other sources. Company-based data retrieved from employers’ personnel files is considered a golden standard, mainly because these data are also used for calculating earnings [5-8].

To identify sick leave measure validation studies, a systematic literature search was performed in PubMed. The search terms “Sick leave”, “absenteeism”, “presenteeism”, “work”, “registries”, “self report”, “questionnaires”, “reproducibility of results”, “validity/validation”, “sensitivity and specificity”, “predictive value of tests”, and “accuracy” were combined in the search. Twelve validation studies were selected. The validity of sick leave data reported in questionnaires or in interviews was studied and analysed against data retrieved from employers’ personnel files [5,6,8-13], insurance companies [9,14-16] and a national social security register [17]. None of the studies validated insurance-based data either from companies or from national social security registers against employers’ personnel files.

The validation studies found discrepancies between self-reported length of absence and insurer-reported compensation payments. The inconsistencies spoke against self-report and were associated with work status, cause of absence as well as personal characteristics [14,15].

A study from the Netherlands found poor agreement between workplace-registered sick leave data and data collected in a questionnaire [11]. The ability of this questionnaire to detect frequency of sick leave spells was reported to have a sensitivity of 55% and specificity of 83%. The remaining nine studies concluded that self-reports yielded acceptable validity [5,6,8-10,12,13,16,17]. The total length of absence was the most widely used measure of sick leave [5,6,8-10,12,13,16], but also prevalence [5,12,17], frequency of spells [5], incidence and duration of sick leave spells [16] were used.

Recall periods ranged from 2 weeks to 4 years and were discussed in several papers [6,9,10,17]. These studies were largely unanimous that shorter recall periods could increase the precision of self-reported sick leave. In two papers, the optimum recall period was recommended to be no longer than 2–3 months to obtain valid measures of absence lengths [6,10].

The Whitehall II study and a Swedish replication of Whitehall II found a worse recollection the longer the absence length [8,13] which indicates that valid self-reporting may be limited to absence of short duration. Finally, relatively high sensitivities were found in studies where data on absence length, frequency and prevalence of absence were provided as pre-specified categorical questionnaire options [5,17].

In Denmark opportunities for register-based research are unique [18]. Data on social public transfer payments, like sickness benefits are registered on a weekly basis in a national register called DREAM [19]. Employees’ sickness benefit paid in excess of two weeks is refundable from the municipality according to the Danish Sickness Benefit Act [20]. DREAM data on sickness benefit has been used for follow-up studies, where return to work (RTW) [21-27], sick leave defined as absence > 2–3 weeks [28-32] and long-term sick leave defined as absence >8 weeks [29,31-40] have been used as endpoints. Study populations have also been defined from the DREAM register [24-26,29,41,42]. One attempt has been made to validate the DREAM register [43]. In this study a random sample of 5,221 Danish citizens were asked about which kind of income they received in a particular week in 2001. According to the DREAM register 82 persons were receiving sickness benefit and of those 38 responded this kind of income; yielding a positive predictive value of 31.7%. The article concluded the DREAM register to be a feasible tool for social and economic research in Denmark. DREAM data on sickness benefit has so far not been validated against workplace-registered sick leave.

### Aim

1) To validate registered sickness benefit data from DREAM against workplace-registered sick leave spells of at least 15 days. 2) To validate self-reported sick leave days during one year against workplace-registered sick leave.

## Methods

### Study population

The study is a cross-sectional study of municipal elder care workers in Aarhus (the second largest city in Denmark). To validate DREAM data a total of 3,554 individuals employed throughout 2006 were identified in the employer’s computerized personnel files. Details on the study population are given in Table 1. Their median age was 47.5 years. The majority were women (95%) who were primarily working as home care workers, assistant nurses and nurses. The male employees worked mainly as home care workers, assistant nurses, maintenance workers, and in the administration. Nineteen percent worked full-time, i.e. 37 hours per week, 70% worked between 36 and 30 hours per week, 7% worked between 29 and 20 hours per week and the remaining 1% worked less than 20 hours per week.

**Table 1:** Description of elder care workers employed throughout 2006 in the municipality of Aarhus (N = 3,554)

		**%**	**n**
**Age in years**, median (interquartile range)		47.5 (39–54)	
**Gender** (% female)		95.3	3,387
**Workplace, local centres:**			
Frederiksbjerg		9.2	326
Hasle-Gellerup-Toftegården		7.5	267
Holme og Skåde		7.4	264
Hørgården og Vejlby		8.1	289
Skelager/Bjørnshøj		4.2	148
Trøjborg og Abildgården		5.6	199
Viby og Rosenvang		6.6	235
North		7.1	251
Northwest		7.5	267
South		8.2	291
Southwest		5.5	197
West		9.1	324
City		7.2	255
Others		6.8	241
**Profession:**			
administration		5.4	190
activity		6.3	222
kitchen and café staff		1.1	39
cleaning		5.5	195
social and health care assistant level I and II		68,0	2419
nurse		10.4	369
maintenance		0.6	21
not defined		1.9	66
remaining staff		0.9	33
**Hours worked per week:**			
37		20.8	738
31-36		40.4	1,434
21-30		34.6	1,229
−20		1.7	59
missing		2.7	94

All subjects in this study were covered by the Danish national health insurance, which provides sickness benefit to those who are unable to work due to disease or injury.

Agreement between self-reported sick leave and workplace-registered data was studied in 2,311 responders to the “Working in eldercare” survey [44] in 2005. The response rate was 73% and 2,139 of these responders were also included in the validation of DREAM data from 2006.

Beside questions about the working environment and health the employees were also asked: “How many sick leave days have you had within the last 12 months?”. The two study populations did not differ regarding gender, age, profession or hours worked per week.

### Measures of sick leave

When an employee needed sick leave, he/she reported absence to his or her immediate superior. An absence form was printed out by the superior, in which the first date of the absence spell was written. This form was handed to the employee, who wrote the last date of the absence spell when he/she returned to work and signed it together with the immediate superior. The dates on the form were entered to a computerised duty roster (from which earnings are calculated) by the immediate superior or a secretary. In each of the 14 municipal eldercare workplaces in Aarhus a number of immediate superiors were responsible for entering absence dates. Simultaneously these absence dates were transferred to the company’s absence records along with a categorization of the absence spell (sick leave, care leave, child’s first sick day etc.). These raw data on absence were retrieved from the workplace records, but only spells related to sick leave were included in the present study. The dates of the first and last day of each sick leave spell were available for each individual. A sick leave spell was counted in calendar days regardless of whether all of these days were work days. Overlapping, consecutive, or duplicate sick leave spells were merged into a single spell.

Sick leave spells entitled to municipal refunding according to the Danish Sickness Benefit Act include the following four categories: 1) spells lasting more than 14 days; 2) spells related to pregnancy; 3) spells due to sick leave in a “flexi job”, i.e. modified job due to permanently reduced workability; or 4) recurrent or anticipated spells due to chronic disease. Spells in categories 2) - 4) shorter than or equal to 14 days were exempted from the employer period and refundable from the first day of absence. For every refundable sick leave spell, we identified the week number in which the spells were seen.

### From workplace absence to sickness benefit compensation

When a workplace-registered absence spell was assumed by the employer to be entitled to tax-financed compensation according to the Danish Sickness Benefit Act, a notification was sent from the human resource department in the municipality of Aarhus to an external private IT-company. This private partner provided data-handling services for the municipality and was responsible for notifying the social services in the sick-listed employee’s municipality of domicile, which grants sick leave compensation benefits. From the social services a form was sent to the sick-listed employee. Within a week detailed information about the particular absence spell, treatment, workplace, current work tasks, education and prospects about the return to work process had to be returned to the social services. This information was exchanged with a social worker from the municipal job centre, engaged in the process of resumption of work. If/when the absence spell was discontinued the social services was notified by the social worker. Data on tax-paid sickness benefit compensation granted in the municipality of domicile are pooled in a central database. This information is further processed and collected in a national register of sickness benefits and maternity payments, which becomes part of the DREAM register.

### Measures of sickness benefit

The DREAM register is administered by The Ministry of Employment [19]. The name is a Danish acronym (Den Registerbaserede Evaluering Af Marginaliseringsomfanget) which translates into “The evaluation of marginalized groups of individuals based on registered social public transfer payments”. The DREAM register includes all Danish citizens with a CPR number who have received social public transfer payments at some point since mid-July 1991. Each person is registered once a week with a code representing the type of reimbursement received that particular week (currently 109 codes are available). Codes are ordered hierarchically; low-ranked codes are overwritten by high-ranked codes, e.g. sickness benefit codes (Table 2). The weekly recordings cover reimbursements of 1 to 5 days of actual workdays lost. By January 2006, data on more than 3.5 million citizens of a total Danish population of 5.4 million had been entered into the DREAM register.

**Table 2:** DREAM codes related to sickness benefit reimbursement

**DREAM-code**	**Various types of sickness benefit**
891	Sickness benefit
892	Sickness benefit while being on part time sick leave
*893*	*Sickness benefit while being unemployed (this code is of no relevance in this study)*
894	Sickness benefit while being re-trained
895	Sickness benefit while being employed under special condition “flexi job”

The weeks of 2006 were isolated in the DREAM register and the 3,554 employees were identified by their CPR number. The weeks in 2006 coded 891, 892, 894 or 895 were identified.

### Statistical analyses

#### Agreement about prevalence of sick leave, aim 1

We used workplace registered sick leave as the reference standard in the comparison of employers’ personnel files and DREAM data on sickness benefit. Whether refundable sick leave and sickness benefit was registered in the employer’s personnel files and DREAM respectively, was addressed in each week of 2006 and cross tabulated. Hence 52 2×2 tables were constructed and sensitivity, specificity and positive predictive value (PPV) of DREAM data were calculated. Logistic regression was applied to obtain single sensitivity probabilities, i.e. the probability of having received sickness benefit registered in DREAM when the workplace data said so. For this analysis, individuals were included as cluster variables to adjust for 48 repeated measurements from each study member. Week 1, 2, 51 and 52 were excluded to avoid possible imprecision due to sick leave carried forward from the previous year and prolonged into the forthcoming year. Gender, dichotomised age (< 41 years), workplace (14 geographically defined categories) and profession (administration, activity, kitchen and café staff, cleaning, social and health care personnel level I and II, nurse and maintenance) were included as explanatory variables.

#### Concordance with sick leave spell durations, aim 1

A receiver-operating characteristic curve (ROC-curve) was used to study the performance of DREAM data on sickness benefit. Weekly cut-off points in DREAM were used to find the optimum discrimination of sick leave spell durations of >8 weeks defined in the workplace register.

The mean duration of the first workplace-registered sick leave spell was calculated as well as for the corresponding number of weeks of sickness benefit reimbursement registered in DREAM. Differences between and averages of these durations formed a Bland-Altman plot and was used to illustrate the relationship between the two durations. The assumptions behind a paired t-test were also appraised from this plot (Wilcoxon´s signed rank test is the non-parametric test equivalent). Included in the analyses were the first spells of sick leave retrieved from the employers’ personnel files and the first registration of sickness benefit in DREAM, whenever agreement about starting week had been established.

Employees who had pregnancy-related sick leave according to the employers’ personnel files at some point in 2006 (95 women) constituted a special group. All analyses were performed both with and without this group and separately for the group as such.

#### Concordance between self-reported and workplace-registered sick leave length, aim 2

Raw data on absence were retrieved from the workplace records, but only spells related to sick leave were included. All sick leave spells 12 months prior to the response date were identified. If a spell encompassed the response date or the date 365 days prior to that, the spell was “shortened” to ensure the duration did not exceed these dates. All sick leave spells were summarised into total length in calendar days. These were compared to the self-reported sick leave days from the questionnaire.

Mean annual length and differences in days were stratified on gender, age, profession and working hours per week. To enhance the comparability to other studies we used some of the same sick leave measures used in a Swedish study [13]. Both measures of sick leave were categorised into: 1) 0 days, 2) 0>days=<7, 3) 7>days=<14, 4) 14 > days < 28, 5) 28>=days<56 and 56 days or more. An expanded 2x2 table was constructed and sensitivity and specificity were calculated.

All analyses were performed in Stata version 11.2.

Approval (2009-41-3828) for conducting this register-based study was obtained from the Danish Data Protection Agency: http://www.datatilsynet.dk/english/.

## Results

### Aim 1

#### Overall prevalence

**Figure 1:**
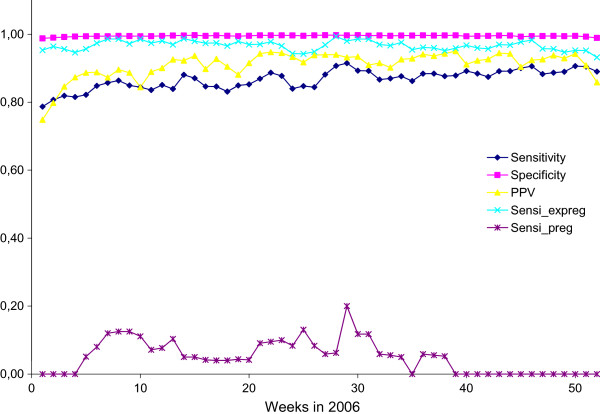
**Sensitivities, specificities and positive predictive values of DREAM-registered sickness benefit.** Sensitivities, specificities and positive predictive values of DREAM-registered sickness benefit calculated in each week in 2006 using workplace data on sick leave as reference standard. Agreement is illustrated with and without pregnancy-related sick leave and separately for this group of women as such.

Separate analyses of sensitivity, specificity and PPV were carried out for each week of 2006 (Figure 1).

From week 5 and onwards, sensitivities and PPVs (including pregnancy-related sick leave) fluctuated steadily. The lowest sensitivity was 79% (95% CI: 71–85) (week 1), the highest sensitivity 92% (95% CI: 86–95) (week 29). The lowest PPV (75%; 95% CI: 68–81) was also found in week 1. The PPV peaked on a few occasions in week 22, 23 and 39 at 95% (95% CI: 91–98). Specificity, however, was consistently close to 100% (95% CI: 98.3-100) throughout the entire year.

Exclusion of the pregnancy-related sick leave substantially increased the sensitivities and caused fluctuation throughout the entire year to be minimal, viz. 93% (95% CI: 89–96) at their lowest and 99% (95% CI: 95–100) at their highest. Specificity and PPV did not change substantially.

Analysis using pregnancy-related sick leave alone showed a very poor performance of DREAM data on sickness benefit with the highest sensitivity reaching only 20% (95% CI: 4–50).

##### Pregnancy-related sick leave excluded

Logistic regression analyses were initially performed without pregnancy-related sick leave. The models were adjusted for 48 repeated individual measurements and showed an overall probability of 96.7% (95% CI: 95.6-97.6) for DREAM data being in concordance with the workplace-derived data on refundable sick leave registered in the employers’ personnel files (sensitivity).

The sensitivity of DREAM data was not statistically significantly different for young female employee compared to older (odds ratio (OR) 0.68; 95% CI: 0.4 - 1.3) or for old and young men (OR 0.9; 95% CI: 0.2 - 5.0). Gender did not affect the sensitivity either: OR was 0.79 (95% CI: 0.5 - 1.3) for men. None of the professions or workplaces either increased or decreased the likelihood of agreement (results not shown).

The DREAM register and the employers’ personnel files for week 3 to week 50 were in agreement that 2,616 of 3,459 employees (75.6%; 95% CI: 74 – 77) were not sick-listed / not receiving sickness benefit and that 789 employees (22.8%; 95% CI: 21–24) were sick-listed and had received sickness benefit, an overall observed agreement of 98.4% (95% CI 98.0-98.8). Twenty-two employees 0.6% (95% CI: 0.4 - 1) were sick-listed according to workplace files, but did not receive sickness benefit according to DREAM. Finally, 32 employees (0.93%; 95% CI: 0.6 – 1.3) were reimbursed according to DREAM, but that figure could not be verified in the employers’ personnel files.

For those 789 employees where both the DREAM register and employers’ personnel files had recorded sick leave, agreement was complete in terms of registered weeks in 557 cases (70.6%; 95% CI: 67–74) and in terms of the number of weeks in 5 cases (0.6%; 95% CI: 0.2 – 2). However, disagreement about which weeks was observed in 173 cases (21.9%; 95% CI: 19–25) where DREAM had registered more weeks than the workplaces and 54 cases (6.8%; 95% CI: 5–9) where the number of workplace-registered sick leave exceeded the weeks of reimbursements in DREAM.

##### Women with pregnancy-related sick leave included

The logistic regression models were also calculated including pregnancy-related sick leave. An overall probability of 87.0% (95% CI: 84.2 – 89.4) of DREAM data being in concordance with the workplace-derived data on refundable sick leave was found. DREAM data was statistically significantly less sensitive among younger than older women with an OR of 0.1 (95% CI: 0.06 - 0.2). Still, sensitivity did not depend on gender; the OR was found to be 1.38 (95% CI: 0.9 - 2.1) for men.

#### Duration of sick leave spells

**Figure 2:**
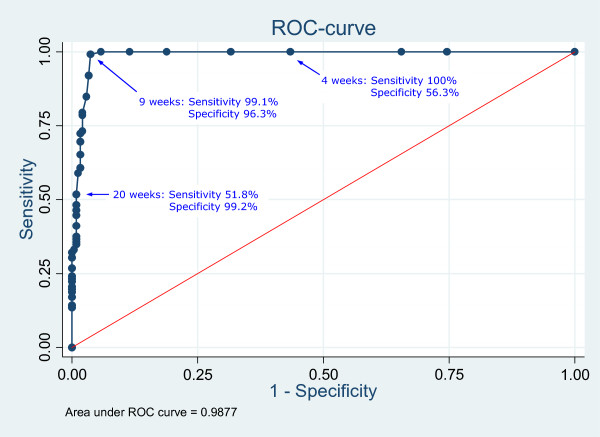
**The accuracy of DREAM to identify workplace-defined sick leave spells of > 8 weeks.** ROC curve showing the sensitivity and specificity corresponding to different choices of cut-off points in DREAM for sick leave spell durations of eight weeks or more defined in the workplace register.

The following results are based on analyses that include the first sick leave spell of 356 employees without pregnancy-related sick leave. The ROC-curve (Figure 2) illustrates that a nine-week optimum cut-off point in the DREAM register was required to discriminate between a sick leave spell duration of eight weeks or more defined in the workplace register. The area under the curve (AUC) was 98.77% (95% CI: 97.8-99.7). This implies that a cut-off point of nine weeks in the DREAM register will correctly identify approximately 99% of workplace-registered sick leave durations of eight weeks or more.

**Figure 3:**
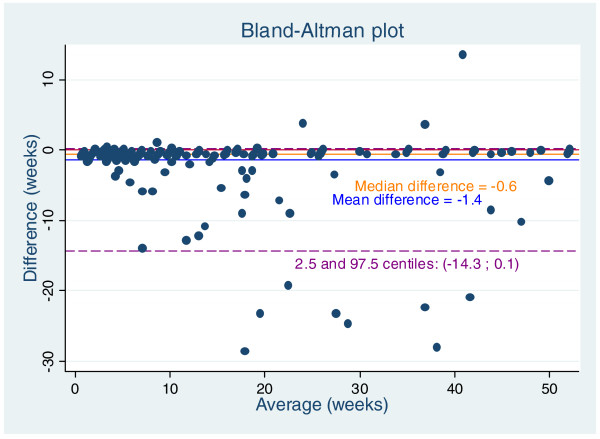
**Bland-Altman plot of workplace-registered sick leave spells and DREAM-registered reimbursement periods.** The average duration of workplace-registered sick leave spells and DREAM-registered reimbursement periods plotted against the difference between these two measures.

The agreement between the two registers in terms of the duration of the first sick leave spell is also illustrated in Figure 3. The mean difference between DREAM data on sickness benefit and the employers’ personnel files was −1.4 (SD: 3.9) weeks, i.e. DREAM data overestimated sickness leave by an average of 1.4 weeks compared with the workplace register. According to the Bland-Altman plot, the difference was not evenly distributed around the y-line = 0 as DREAM data overestimated the workplace-registered sick leave in most cases. Furthermore, the difference was not independent of the average value; thus, clustering of dots illustrates that the shorter average duration, the less difference between the two registers. The average difference between the two registers was statistically significantly different from zero (p < 0.001).

### Aim 2

#### Total annual sick leave length

The self-reported mean annual length of sick leave was lower than workplace-registered sick leave. For women the mean difference between workplace-registered and self-reported sick leave was 4.3 (95% CI: 3.4-5.2) days and for men 4.8 (95% CI: 0.4-9.2) days. The youngest age group (19–29 years) recalled their absence with the lowest precision with a mean difference of 7.2 (95% CI: 3.1-11.2) days compared to the age group of 40–49 years with a mean difference of 3.9 (95% CI: 2.7-5.1) days. Totally, 1,063 individuals underestimated their sick leave, 662 individuals recalled precisely and 586 individuals overestimated their sick leave.

When workplace-registered and self-reported sick leave lengths were categorised as shown in Table 3; 518 individuals underestimated their sick leave, 1,502 recalled their sick leave accurately and 251 eldercare workers overestimated their sick leave. The highest agreement was found in the categories 0, 0–7 and >56 days, where the responders were able to accurately recall annual lengths in 85.3 (95% CI: 81.4 – 88.6) %, 78.1 (95% CI: 75.3 – 80.8) % and 58.5 (95% CI: 51.1 – 65.6) % of the cases, respectively. In total, of those having a least one sick leave day according to the workplace register (n = 1,910), 1,805 individuals also reported so themselves, which was equivalent to a sensitivity of 94.5 (95% CI: 93.4- 95.5) %. Among the eldercare workers who did not have any sick leave days (n = 401) 342 individuals reported so giving a specificity of 85.3 (95% CI: 81.4 – 88.6) %. The sensitivity of recalling having had >=28 or >=56 sick leave days was 64.7 (95% CI: 59.4 – 69.7) % and 58.5 (95% CI: 51.1 – 65.6) %, respectively. The corresponding specificities were 98.3 (95% CI: 97.6 – 98.8) % and 98.9 (95% CI: 98.3 – 99.3) %.

**Table 3:** Workplace-registered and self-reported sick leave days among municipal eldercare workers in a 12 month period

	**Workplace-registered weeks**												
	**0 days**		**0 > days = <7**		**7 > days = <14**		**14 > days < 28**		**28 > =days < 56**		**> = 56 days**		
**Self-reported**													
**Weeks**	**n**	**%**	**n**	**%**	**n**	**%**	**n**	**%**	**n**	**%**			
0 days	342	**85.3**	88	9.7	9	2.4	3	1.1	3	1.9	2	1.1	447
0 > days = <7	47	11.8	711	**78.1**	146	38.7	37	13.5	15	9.4	14	7.5	970
7 > days = <14	6	1.5	96	10.6	179	**47.5**	111	40.4	19	11.9	9	4.8	420
14 > days < 28	3	0.8	10	1.1	39	10.4	102	**37.1**	49	30.6	12	6.4	215
28 > =days < 56	3	0.8	2	0.2	3	0.8	18	6.6	58	**36.3**	41	21.8	125
> = 56 days	0	0.0	3	0.3	1	0.3	4	1.5	16	10	110	**58.5**	134
	401		910		377		275		160		188		2311
	**Any sick leave**								**> = 28 days**		**> = 56 days**		
Sensitivity	94.5% *								64.7%		58.5%		
Specificity	85.3%								98.3% **		98.9% ***		

Percentages in bold shows perfect agreement between self-reported and workplace-registered sick leave weeks.

* (910–88) + (377–9) + (275–3) + (348–5) / (910 + 377 + 275 + 348).

** (401–3) + (910–5) + (377–4) + (275–22) / (401 + 910 + 377 + 275).

*** (401–0) + (910–3) + (377–1) + (275–4) + (160–16) / (401 + 910 + 377 + 275 + 160).

## Discussion

Our study showed an excellent agreement between workplace registered sick leave and DREAM registered sickness benefit compensation. Except for pregnancy-related sick leave, the DREAM register identified workplace-registered spells exceeding 14 days with very high sensitivity and excellent specificity. To identify sick leave spells beyond eight weeks, the optimal cut-off point in the DREAM register was nine weeks. On average, DREAM data overestimated the workplace-specified duration of sickness spells by 1.4 weeks. The ability to recall accurately declined the higher number of workplace-registered absence days. This was apparent even with short lengths; only 47.5 (95% CI: 42.3 – 52.7) % and 37.1 (95% CI: 31.4 – 43.1) % recalled accurately that they had had 7–14 days or 15–28 days of sick leave, respectively.

### Other validity studies

To our knowledge, no previous study has attempted to validate a national social security register against workplace-registered sick leave so comparison with other studies is not possible.

Studies providing self-reported data on total absence length, frequency and prevalence as pre-specified categorical options reported relatively high sensitivities (range 79-91% with workplace registers as reference standard) [5,13,17]. The sensitivity of 91% was found when responses were categorised in having had at least one day of absence within one year, but the specificity was low (74%) [13]. Regarding absence of more than 28 days within one year the sensitivity was 67% and the specificity was 98%. We were able to reproduce the results reported in the study by Voss et al. in our study. In the validation of DREAM data on sickness benefit, both sensitivity and specificity were high although we adopted a much stricter requirement of sick leave registration within the same week than the criterion of sick leave within the same year used in studies using self-reported sick leave. Other studies requesting more information on sick leave reported lower sensitivities. Thus, a decline in sensitivity from 79% to 64% [17] and from 79% to 13% [5] were seen when additional information about diagnosis was required.

In this study, we analysed the validity of DREAM registered sick leave spell duration. A frequent topic addressed in validation studies is the respondent’s ability to precisely recall sick leave duration within a specified timeframe. Recall periods of down to two months have been shown to produce discrepancies between workplace data and self-reported duration of absence in approximately 13% of the cases. This percentage increased to approximately 50% when the recall period was extended to 12 months [6]. In our study population the ability to recall absence lengths accurately was found in 662 cases (28.7%). Approximately 30% perfect agreement between self-report and workplace registered data among female responders was also found in the Whitehall II study [8]. For comparison, DREAM data was in perfect agreement with workplace data in 69.7% of the cases on which weeks were reimbursed and on the respondent being on sick leave. However, DREAM data overestimated the spell duration by a mean of 1.4 weeks. Overestimation was less pronounced at shorter spells. Due to the registration procedure in the DREAM register, an expected and systematic overestimation can explain some of the variation seen in our study because one week in the DREAM register covers reimbursement of 1 to 5 days of actual workdays lost.

The agreement between DREAM data on sickness benefit and workplace data did not depend on the women’s age. This changed when pregnancy-related sick leave was included in the analyses because of the combined effect of being young and therefore more prone to be pregnant. Furthermore, the age dependency could not be found among the male employees. Because pregnancy-related sick leave is recoded from sickness benefit to maternity payment, sick leave among young women is underestimated and caution is advised when analysing sick leave in this age group of females.

Male employees seemed to give more valid self-reported data than women [8,9,13]. The massive female employee domination in the public sector and in our study population hampers firm conclusions about potential gender differences in terms of diagnostic accuracy of sick leave in our data.

Prior validation studies have established that the ability to correctly recollect the duration of absence decreases with the duration of the absence [6,8,13]. This was also the case regarding our self-reported data. A longer absence length was associated with increased discrepancies between workplace-registered and self-reported sick leave. Under- or over reporting was evident for 1,063 and 586 individuals, respectively. When using categorisation of sick leave duration the number of responders who underestimated their sick leave declined to 518. Still the ability to recall sick leave length accurately decreased with increased lengths of absence.

#### Methodological considerations

The present study included an entire population of eldercare workers in Aarhus, employed throughout 2004 and 2005 or 2006. In the validation of DREAM data nobody was lost to follow-up, thus selection bias was not an issue. In the validation of the self-reported sick leave, a total of 3,147 eldercare workers were employed throughout 2004 and 2005, but only 2,311 (73%) responded. Responders and non-responders had a mean workplace registered annual absence length of 2.5 (SD: 5.0) weeks and 4.2 (SD: 8.0) weeks, respectively (results not shown). We expect those with increased absence lengths to recall with less precision compared to those with shorter lengths. Therefore, some selection bias may have been present and caused overestimated results regarding agreement - in line with findings of Burdorf et al. [5]. This is a major disadvantage of self-reports as compared with register data.

The large number of participants yielded a high power reflected in precise measurements and tests. Yet, the population was strongly dominated by women and the power to reveal gender difference was therefore questionable.

There was a lower agreement between DREAM data and workplace data in the measures obtained at the beginning and towards the end of 2006 than during the remaining part of the year. This can be explained by different registration procedures. We do not expect excluding these weeks to have caused information bias because having sick leave in these particular weeks would appear at random.

The registrations in both the DREAM register and the employers’ personnel files are based on manual entry and human error might explain the disagreement between the two registers on 1.55% of the employees. This validation study was based on data from one municipality. Even if manual entry of sick leave data was done by a number of different administrative employees working on different geographical settings and workplaces within the same municipality, the procedure was most likely systematised in a way that differs from that used in other companies. Future research projects should repeat the validation study using data from workplaces from the private sector and state institutions. The municipal health care workers account for approximately 20% of the total municipal workforce of 500,000 persons in Denmark [45].

#### Possible implication of the DREAM data validation

Until 2011, we found 22 studies that used data retrieved from DREAM for sick leave research. Studies defined the study population from the DREAM register as those who had received sickness benefit for at least two to three weeks before baseline [24-26,29,41,42]. The DREAM register correctly registered sickness benefit reimbursements in 96.7% of those weeks that were also verifiable in the employers’ personnel files in our study. In general some bias should be expected because the DREAM register does not register sick leave as such but sickness benefit and other disability benefits. However, we believe that the agreement between sickness benefit and sick leave in our study is reasonable and that it supports the use of DREAM data as a selection tool to identify sick leave periods with little or no selection bias.

DREAM data used for follow-up studies [29,31-40] defined long-term sick leave as absence >8 weeks. When a cut-off point of nine DREAM-registered weeks of sickness benefit was chosen, the ability to discriminate workplace-registered spells of >8 weeks was optimal. However, from our point of view, this misclassification was non-differentiated which would tend to bias the results toward the null hypothesis.

In comparative register studies on sickness absence where the DREAM register is considered for use; it will be of great importance to recognise the recoding of pregnancy related sickness absence into maternity benefits, because it is reducing the validity in fertile-aged women.

Reliability of self-reported sickness absence declines with increasing length of absence and for studying absence beyond the period paid by the employer, national registers seems preferable to self-reported data if reliable personnel files are not available.

## Conclusion

DREAM data on sickness benefit is a valid measure of sick leave spells lasting at least 15 days among Danish municipal eldercare workers. Self-reported annual sickness absence shows good agreement for total lengths not exceeding 1 week. DREAM offers valid, objective measurements and imprecision due to recall errors is thus avoided. Self-reported sick leave becomes more imprecise when number of absence days increases, but the sensitivity and specificity are acceptable for lengths not exceeding one week. However, the duration of sick leave spells from the DREAM-register should be interpreted cautiously. DREAM data is not valid in relation to pregnancy-related sick leave.

## Competing interests

The authors declare that they have no competing interests.

## Authors’ contributions

CMS conceived the study, carried out statistical analyses and drafted the manuscript. NTA and NF carried out and / or supervised the statistical analyses. All authors participated in the design of the study, helped to draft the manuscript and interpreted the results. All authors have read and approved the final manuscript.

## Funding

This work was partly supported by The Danish Working Environment Research Fund [20080016279/3].

## Pre-publication history

The pre-publication history for this paper can be accessed here:

http://www.biomedcentral.com/1471-2458/12/661/prepub
